# Auricular Composite Grafts Conforming to Nasal Cavity Anatomy Enable Functional and Aesthetic Reconstruction of Alar Full-Thickness Defects

**DOI:** 10.7759/cureus.94007

**Published:** 2025-10-07

**Authors:** Yuto Yamamura, Kazuyasu Fujii, Chisa Nakashima, Shunya Usui, Kazutoshi Nishimura, Atsushi Otsuka

**Affiliations:** 1 Dermatology, Kindai University, Osaka, JPN; 2 Dermatology, Kindai University Hospital, Osaka, JPN

**Keywords:** auricular composite graft, full-thickness defect, intranasal lining reconstruction, nasal ala repair, nasolabial flap, ­reconstructive surgery

## Abstract

The nasal ala is an anatomically complex region composed of external skin, structural support, and internal lining, each of which must be reconstructed to achieve both functional and aesthetic outcomes. Conventional multilayered reconstructions often require technically demanding and staged procedures, imposing a considerable burden on both surgeon and patient. We report a male in his 60s with a sebaceous carcinoma of the right nasal ala. Following tumor excision with a 5-mm margin, a full-thickness alar defect was reconstructed in a single stage. An auricular composite graft, harvested in a shape conforming to the defect, was used to restore the mucosal lining and structural support, while a transposition nasolabial flap provided external cover. The graft survived completely and maintained a natural intranasal contour, and the external bulk gradually improved over time, resulting in an acceptable cosmetic outcome without the need for revision surgery. The auricular donor site healed uneventfully without deformity. Auricular composite grafts are traditionally used for reconstructing the alar rim or columella; however, to our knowledge, their application to the intranasal lining has not been reported. In this case, the graft provided both mucosal stability and structural support, demonstrating the morphological adaptability of auricular tissue. This technique allowed relatively simple and reliable single-stage reconstruction of a full-thickness alar defect. Auricular composite grafts tailored to the defect morphology may represent a practical and versatile option for intranasal lining and structural reconstruction in selected patients.

## Introduction

The nasal ala is an anatomically complex region composed of three distinct layers (cutaneous cover, structural support, and vestibular lining) that together maintain both airway patency and nasal symmetry, making it a functionally and aesthetically critical subunit of the face [1,2]. When full-thickness alar defects occur, successful reconstruction requires the restoration of all three layers to prevent alar collapse, nostril stenosis, and cosmetic deformity [3].

Conventional reconstructive strategies often involve staged procedures using combinations of local or regional flaps. For example, interpolated cheek or forehead flaps combined with cartilage grafts have been widely employed to restore the external cover and provide structural stability [4,5]. Similarly, septal mucosal hinge flaps or nasolabial turnover flaps have been used to replace the intranasal lining [6,7]. Although these approaches can achieve reliable outcomes, they are technically demanding and frequently require multiple operations, which may place a significant burden on both surgeon and patient [4,6].

Auricular composite grafts, which contain both skin and cartilage, have traditionally been applied to reconstruct small defects of the alar rim or columella [8,9]. Their intrinsic three-dimensional morphology and structural rigidity make them particularly suitable for maintaining nostril contour. However, restoration of the intranasal lining in full-thickness alar defects remains technically demanding, and the potential use of auricular composite grafts for this purpose has not been explored. However, to our knowledge, their application as an intranasal lining in the context of full-thickness alar reconstruction has not been reported.

Herein, we present a case of sebaceous carcinoma of the nasal ala that resulted in a full-thickness alar defect following oncologic resection. The defect was reconstructed in a single stage using an auricular composite graft tailored to the intranasal contour for lining and support, combined with a nasolabial flap for external coverage. This case highlights the morphological adaptability of auricular composite tissue and underscores its potential as a practical option for selected patients requiring single-stage reconstruction of complex alar defects.

## Case presentation

**Figure 1:**
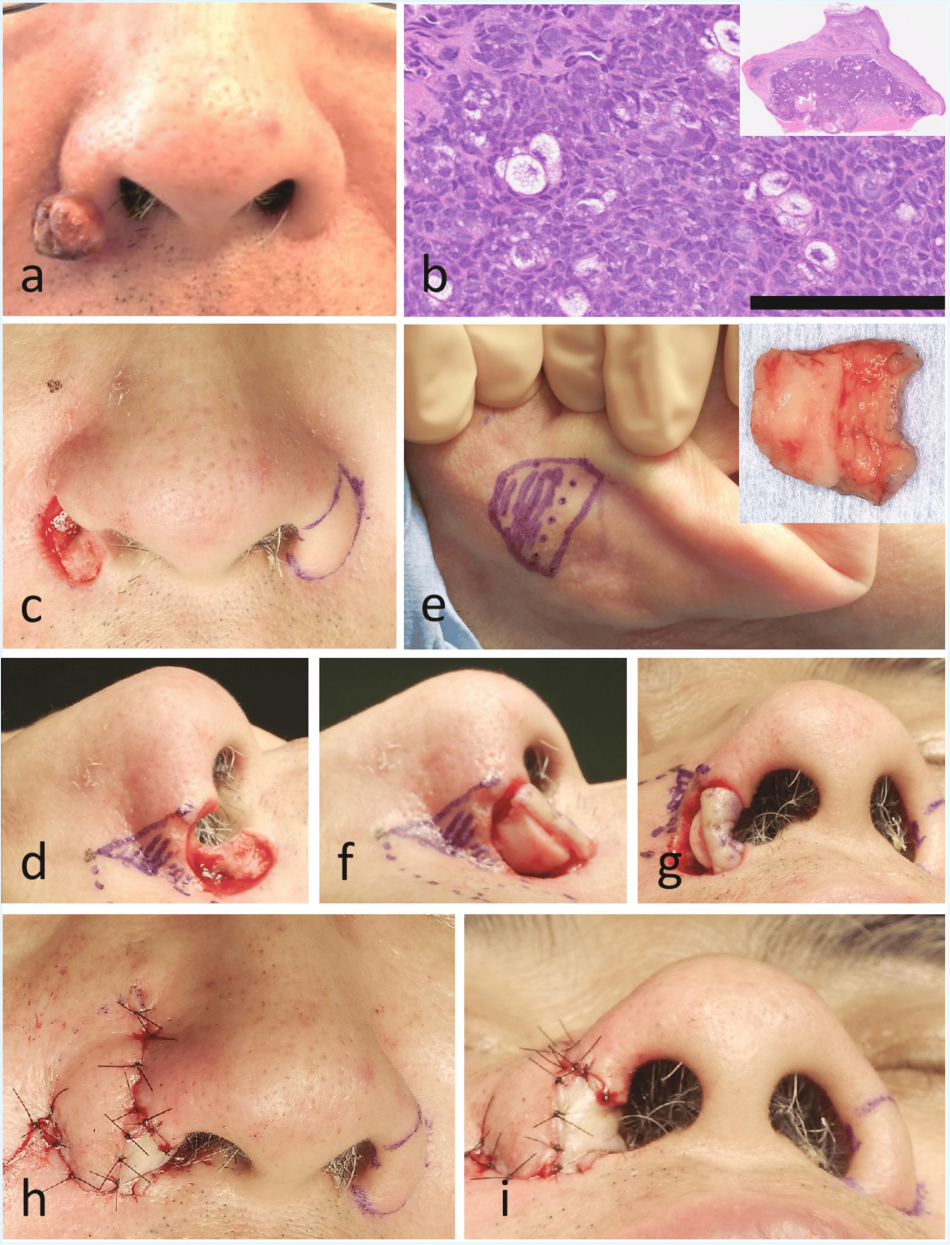
Clinical, histopathological, and intraoperative findings. (a) A 1.5-cm sebaceous carcinoma on the right nasal ala. (b) Low-power view (inset) shows lobules of tumor cells beneath the epidermis. Higher magnification (hematoxylin–eosin, scale bar = 100 μm) demonstrates tumor cells with vacuolated cytoplasm and nuclear atypia, consistent with sebaceous differentiation. (c, d) Full-thickness alar defect following tumor excision with a 5-mm margin. (e) Design of the auricular composite graft for the mucosal lining and structural support. Both auricles were examined preoperatively, and a donor site conforming to the shape of the nasal defect was selected. The shaded area indicates the portion including cartilage, while the unshaded area indicates skin only. (f, g) Placement of the auricular composite graft into the defect, restoring a natural intranasal contour. The skin-only portion of the graft was folded to reconstruct the nasal floor. (h, i) The external cover was reconstructed with a transposition nasolabial flap elevated along the right nasolabial fold. A relatively large flap was raised to ensure sufficient perfusion of the underlying graft.

A male in his 60s presented with a gradually enlarging reddish nodule on the right nasal ala that had first appeared four years earlier. On examination, a 1.5-cm reddish pedunculated mass was observed on the right alar region (Figure 1A). Incisional biopsy confirmed a diagnosis of sebaceous carcinoma (Figure 1B).

The lesion was excised under general anesthesia with a 5-mm margin, resulting in a full-thickness alar defect (Figure 1C). An auricular composite graft consisting of skin and cartilage was harvested from the posterior surface of the right auricle (Figure 1D). The graft was tailored to conform to the shape of the alar defect and placed to reconstruct the mucosal lining and provide structural support (Figure 1E). The external cover was reconstructed with a transposition nasolabial flap elevated along the right nasolabial fold (Figure 1F). The auricular skin component was compressed and fixed within the nasal cavity using a cotton ball. Histopathological examination of the excised specimen confirmed sebaceous carcinoma with tumor-free margins.

**Figure 2:**
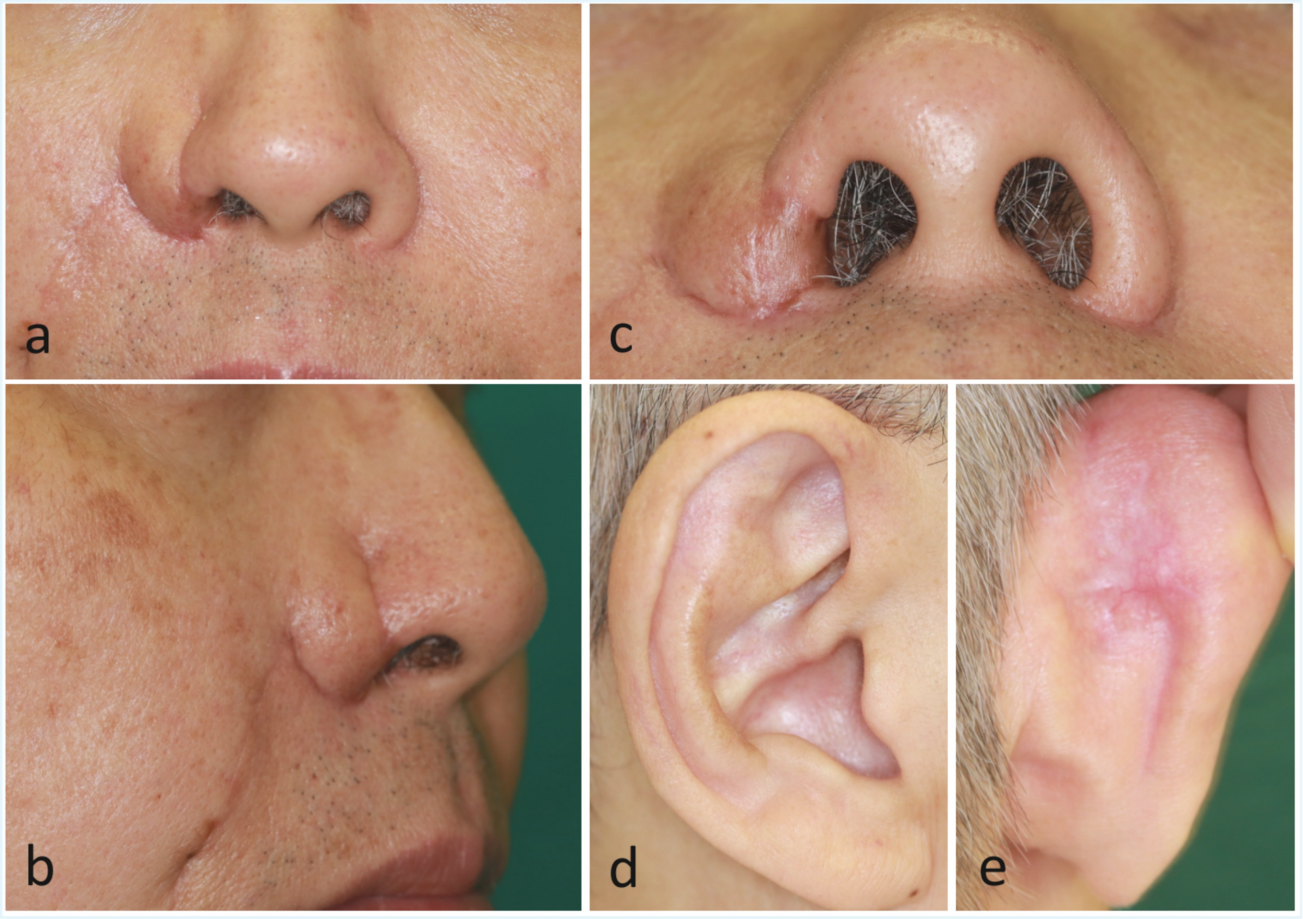
Postoperative course of the nose and the auricular donor site at six months. (a) Frontal view showing residual bulkiness of the external cover. (b) Lateral view showing a somewhat bulky external cover. (c) Basal view demonstrating a stable intranasal contour. (d) Anterior view of the auricle demonstrating good healing of the donor site. (e) Posterior view of the auricle showing no deformity.

At six months postoperatively, the external cover remained somewhat bulky (Figures 2A, 2B), but the cosmetic outcome was acceptable, and the patient did not undergo revision surgery. The auricular composite graft demonstrated good survival, and the intranasal contour was well preserved (Figure 2C). The donor site of the auricle healed uneventfully without deformity (Figures 2D, 2E).

## Discussion

In the present case, a full-thickness alar defect following oncologic resection was reconstructed in a single stage using an auricular composite graft for intranasal lining and structural support, combined with a nasolabial flap for external cover. At six months postoperatively, the graft showed stable survival and maintained airway patency and contour, achieving outcomes comparable to those of conventional multi-staged reconstructions but with less operative burden. This approach avoided multi-staged procedures while maintaining both nostril contour and airway patency.

Compared with conventional reconstructions using interpolated cheek or forehead flaps [4,5], the present technique was simpler and less invasive, yet still achieved satisfactory functional and cosmetic outcomes. From a dermatologic surgery perspective, this is particularly valuable because many patients with cutaneous malignancies of the nasal ala are elderly and may not tolerate staged procedures well.

Auricular composite grafts have been described for reconstruction of the alar rim or columella [8,9]. Composite grafts have also been used in combination with nasolabial flaps to reconstruct full-thickness alar defects, where the flap was hinged intranasally to provide vascular support for the graft [10]; however, to our knowledge, their use as the sole material for both lining and structural support has not been reported. In our case, the graft provided both mucosal stability and mechanical rigidity as the intranasal lining and structural support, highlighting the morphological adaptability of auricular tissue. Importantly, the graft was harvested in a shape conforming precisely to the intranasal defect, which allowed restoration of the natural nasal contour without donor-site deformity. This expands the reconstructive options available to dermatologic surgeons who frequently manage alar tumors.

For dermatologic surgeons, this case highlights that reconstructive planning should be considered an integral part of tumor management. Auricular composite grafts may offer a practical, single-stage option in selected patients, particularly when patient comorbidities or tolerance make staged procedures less feasible.

Nevertheless, limitations exist. The survival of composite grafts is generally restricted to defects up to approximately 1-1.5 cm in width [8]. For larger alar defects, additional measures to ensure graft vascularity, such as hinging the nasolabial flap intranasally as previously reported [10], or multi-staged flaps such as forehead flaps [3,4], may be necessary. Moreover, a longer follow-up is needed to assess the durability of the contour and airway patency. Future comparative studies would clarify the precise indications for composite graft-based versus flap-based reconstructions.

## Conclusions

In summary, this case demonstrates that auricular composite grafts can serve not only as structural support but also as intranasal lining in full-thickness alar reconstruction. The morphological adaptability of auricular tissue enabled precise restoration of the nasal contour without donor-site deformity. For dermatologic surgeons treating cutaneous malignancies of the nasal ala, this represents a practical, single-stage option that balances oncologic safety, functional preservation, and cosmetic acceptability. Moreover, the simplicity and reproducibility of this approach suggest that it may be applicable in a wide range of clinical settings, particularly for elderly patients or those who may not tolerate multi-staged procedures. Further accumulation of cases and longer follow-up will help clarify the durability of this technique and refine its indications.
